# Assessment of the Most Popular Educational Content in Arabic Available Through Four Social Media Applications in Saudi Arabia That Explains Idiopathic Clubfoot Deformity

**DOI:** 10.7759/cureus.76976

**Published:** 2025-01-05

**Authors:** Omar B Alammari, Omar Odeh, Rakan Alotaibi, Mohammed Zamzam, Abdullah S Alghamdi

**Affiliations:** 1 Surgery/Ophthalmology, King Khalid University Hospital, College of Medicine, King Saud University, Riyadh, SAU; 2 General Practice, College of Medicine, King Saud University, Riyadh, SAU; 3 Internal Medicine, College of Medicine, King Saud University, Riyadh, SAU; 4 Orthopaedics, King Saud University, Riyadh, SAU; 5 Orthopaedic Surgery, Prince Sultan Military Medical City, Riyadh, SAU

**Keywords:** claw foot, clubfoot, instagram, social media, tik tok, x (formerly twitter), youtube videos

## Abstract

Background

With the growing availability and popularity of social media, it is essential to assess the quality and accuracy of the information available about health conditions. This study aims to evaluate the quality of Arabic content related to clubfoot on four social media platforms: YouTube, TikTok, X (formerly known as Twitter), and Instagram. Additionally, it seeks to understand how caregivers of children with clubfoot can utilize social media.

Methods

This study involved a qualitative analysis conducted over six months, focusing on the Arabic clubfoot community on social media. Data collection included comments, posts, and short clips, which were recorded in a Google Sheet. The study was approved by an Institutional Review Board (IRB), and all procedures adhered to the guidelines set forth in the Declaration of Helsinki. Data analysis utilized descriptive statistics and bivariate analysis, with results presented in tables.

Results

The study identified a total of 14 videos and posts on TikTok, nine posts on X (formerly Twitter), 18 videos and posts on YouTube, and 10 posts on Instagram. Notably, TikTok video 2 garnered the highest engagement, receiving 76,000 views and 561 likes. The top-performing post on X achieved 140 likes. YouTube video 3 received 31,239 views along with 479 likes, while Instagram post 7 attracted the highest engagement, with 76 likes and 44 comments.

Conclusion

Overall, the quality of Arabic content regarding clubfoot on YouTube, TikTok, X, and Instagram was found to be generally poor. Healthcare organizations should consider utilizing social media platforms to promote awareness and education about clubfoot treatment and management.

## Introduction

Clubfoot is a frequently occurring congenital foot disorder, affecting about 2.2 in every 1,000 infants in Saudi Arabia [1]. If not addressed early, it can lead to complications like infections, challenges in wearing regular footwear, ongoing foot pain, and walking abnormalities [2]. Families dealing with clubfoot often experience emotional stress as they navigate the process of repeated casting and extended periods of orthotic support [3].

Approximately 98.6% of people in Saudi Arabia have access to the Internet [4], highlighting a significant shift in how health-related information is obtained. With the rise of social media, more than 5.35 billion people worldwide can now share insights and experiences [5]. By August 2024, YouTube had reached 2.5 billion active users [6], followed by Instagram, TikTok, and Twitter (now X), with 2.4 billion [7], one billion [8], and 368 million users [9], respectively.

Patients and caregivers frequently use social media platforms to seek medical advice, share their health experiences, and explore different treatment options. For parents of children with clubfoot, these platforms offer a valuable source of information on care and treatment [10,11].

The goal of this study is to evaluate the quality of Arabic content available on YouTube, TikTok, Twitter (now X), and Instagram regarding clubfoot and to examine how caregivers of affected children can use these platforms to find useful information.

## Materials and methods

The study is a descriptive qualitative-based study conducted over a six-month period to evaluate the quality of Arabic content on the most popular social media platforms (TikTok, X [Twitter], Instagram, and YouTube) regarding clubfoot and to assess the utility of these platforms for caregivers of children with clubfoot. Ethical approval was secured from the Biomedical Ethics Committee of King Saud University, Riyadh, Saudi Arabia (approval number: E-23-8308). The Arabic keywords searched were [(اﻟﻘدﻣﯾن ﺣﻧف) AND (اﻟﺣﻧﻔﯾﺔ اﻟﻘدم) AND (اﻟﻣﺧﻠﺑﯾﺔ اﻟﻘدم)]. These Arabic keywords were translated to the following English keywords: [(clubfoot) AND (claw foot)]. All videos and posts in Arabic that matched the Arabic keywords were included in the study. Duplicated content with fewer than 10 views or advertisements aimed at hospitals or healthcare workers were excluded. The search was based on videos and posts published on these four platforms. A total of 51 videos and posts (18 on YouTube, 14 on TikTok, 10 on Instagram, and nine on X [Twitter]) were identified, filtered, and scanned. They were then analyzed, and data were extracted.

After identifying videos and posts, a content validation check was conducted by a professor of pediatric orthopedic surgery to ensure the accuracy of the data used and provide a better representation of the results obtained.

The contents were evaluated using two scoring systems. Initially, the Global Quality Score (GQS), a five-point scale, was used for both videos on TikTok, Instagram, and YouTube, as well as the text content on X (Twitter) to determine its accuracy and quality. This score is considered a validated quality measure ranging from 1 (very unlikely to be of any use to patients) to 5 (excellent quality, and highly useful to patients).

Then, either the DISCERN or the modified DISCERN score was applied only to videos to assess their reliability. The modified DISCERN is considered a high measure of reliability. It has a potential total score of five points, with higher scores indicating higher reliability. DISCERN, on the other hand, is a reliable and valid instrument for judging the quality of written consumer health information. It is used to identify issues of potential bias, relevance, and content currency. It contains 16 questions, rated on a scale of 1-5, with the following grading: excellent (63-80 points), good (51-62 points), fair (39-50 points), poor (27-38 points), or very poor (16-26 points). It is designed for use by consumers and health professionals to rate the overall quality of a publication.

After applying the appropriate scores to the content, the following data were collected: name of the platform, type of content that the post included (which could be more than one option) (definition, outcome, signs and symptoms, risk factors, investigation, evaluation, management, and patient sharing experience), the URL link, number of views, likes, and comments, date of publication, and video length. Data about the type of uploader (either individual or organization), account verification status, and location were also collected.

A face validation check was also performed to evaluate whether the questionnaire works as claimed and whether it serves the purpose of the current study.

The frequency and percentage were calculated for demographic data, outcomes, signs and symptoms, risk factors, and others using the SPSS (Statistical Package for Social Sciences) Statistics, version 24 (IBM Corp, Armonk, NY). The mean and standard deviation were also used to describe the quantitative and categorical variables.

The relationship between the mean values and the categorical study variables was calculated using bivariate analysis (Student’s t-test for independent samples, one-way analysis of variance [ANOVA] followed by a post-hoc test for the quantitative outcome variables). In addition, Pearson’s chi-square test was used to test the association between the categorical study variables and the outcome variables, and the odds ratios were used as a measure of association between two categorical variables. The analyzed data were presented in tabular format. A p-value < 0.05 was considered the accepted level of statistical significance during the study.

## Results

As observed in Table 1, YouTube emerges as the dominant platform with the highest content share (18; 35.29%), underscoring its wide reach and effectiveness in engaging users. The predominance of videos (38; 74.51%) as the leading media type highlights their visual appeal and ease of consumption, making them more engaging compared to static images. A high proportion of content related to management information (40; 78.43%) and the significant regional representation from Saudi Arabia (13; 40.62%) suggest that localized and specific topics have higher traction. This pattern reflects user preferences and the effectiveness of targeted content strategies in the region.

**Table 1:** Frequency and percentage distribution of platforms, media types, and content characteristics

Variables		Freq	Percent
Platform	Instagram	10	19.61
	TikTok	14	27.45
	Twitter	9	17.65
	YouTube	18	35.29
Media type	Pictures	13	25.49
	Video	38	74.51
Types	FALSE	49	96.08
	TRUE	2	3.92
Definition	FALSE	18	35.29
	TRUE	33	64.71
Outcomes	FALSE	20	39.22
	TRUE	31	60.78
Signs and symptoms	FALSE	30	58.82
	TRUE	21	41.18
Risk factors	FALSE	24	47.06
	TRUE	27	52.94
Evaluation	FALSE	40	78.43
	TRUE	11	21.57
Management	FALSE	11	21.57
	TRUE	40	78.43
Sharing experience	FALSE	39	76.47
	TRUE	12	23.53
Comments	No	12	23.53
	Yes	39	76.47
Vids length (min)	1-3	10	27.03
	10-12	2	5.41
	4-6	3	8.11
	7-9	5	13.51
	<1	17	45.95
Type of the uploader	Individual	24	47.06
	Organization	27	52.94
Verification status	No	47	92.16
	Yes	4	7.84
Location	Egypt	9	28.12
	India	1	3.12
	Iraq	3	9.38
	Jordan	3	9.38
	Morocco	1	3.12
	Qatar	1	3.12
	Saudi Arabia	13	40.62
	UAE	1	3.12

Engagement metrics indicate a median of eight comments and 45 likes, suggesting moderate user interaction. With a median of 8,030.5 views, content achieves substantial reach. The DISCERN scores, with medians of 2 (total) and 3 (Global Quality Score (GQS)), reflect consistent but moderate informational quality. These findings suggest a balance between audience engagement and the perceived value of the information provided. Variations in engagement metrics highlight the need for platform-specific strategies to maximize user interaction. For picture-based content, the mean DISCERN score of 47.5, coupled with a standard deviation of 21.53, indicates moderate informational quality with notable variability. The relatively high standard deviation reveals inconsistencies in the quality of picture-based content, which may challenge user trust and content reliability. These findings emphasize the need for improved curation of visual content to ensure consistency and maintain credibility (Table 2).

**Table 2:** Distribution of engagement metrics, GQS and DISCERN scores: median, quartiles, mean and standard deviation GQS: Global Quality Score.

	Number of comments	Number of Likes	Number of views	Total DISCERN score (Video)	GQS	Total DISCERN score (Pictures)
Median	8	45	8030.5	2	3	-
lower quartile	2	19	2180	1	2	-
upper quartile	40	127	16800	3	3	-
Mean	-	-	-	-	-	47.5
SD	-	-	-	-	-	21.53

Table 3 highlights the statistical analyses of associations between platform types, engagement metrics and DISCERN scores. 

**Table 3:** Statistical analyses of associations between platform types, engagement metrics, and DISCERN Scores GQS: Global Quality Score.

Section	Variables	Obj	Median	Rank Sum	p-value	Test	Spearman’s Rho	Pearson Correlation
4A Kruskal-Wallis test for association between platform type and number of comments	Platform							
	Instagram	7	3	118.50	0.0041	13.289	NA	NA
	TikTok	11	5	178.00	0.0041	13.289	NA	NA
	Twitter	5	2	44.50	0.0041	13.289	NA	NA
	YouTube	16	33	439.00	0.0041	13.289	NA	NA
4B Kruskal-Wallis test for association between platform type and number of likes	Platform							
	Instagram	10	19	167.50	0.0047	12.975	NA	NA
	TikTok	14	49.5	393.50	0.0047	12.975	NA	NA
	Twitter	9	9	151.00	0.0047	12.975	NA	NA
	YouTube	18	95	614.00	0.0047	12.975	NA	NA
4C Kruskal-Wallis test for association between uploader type and GQS, and total DISCERN score for video content								
	Total DISCERN Score (Video Content) Individual	19	2	330	0.2113	-1.250	NA	NA
	Total DISCERN Score (Video Content) Organization	19	2	441	0.2113	-1.250	NA	NA
	GQS - Individual	24	-2	544.5	0.1178	-1.554	NA	NA
	GQS - Organization	27	3	781.5	0.1178	-1.554	NA	NA
4D Spearman's rank correlation between engagement metrics and DISCERN scores for video content								
	Numbers of likes - GQS	51	NA	NA	0.6797	NA	0.0590	NA
	Numbers of likes - Total DISCERN score (video)	38	NA	NA	0.5589	NA	-0.0974	NA
	Numbers of comments - GQS	39	NA	NA	0.6970	NA	-0.0641	NA
	Number of comments - Total DISCERN score (video)	30	NA	NA	0.7406	NA	-0.0626	NA
	Numbers of views - GQS	34	NA	NA	0.4603	NA	0.1305	NA
	Numbers of views - Total DISCERN score (video)	31	NA	NA	0.5919	NA	-0.0996	NA
4E Pearson correlation and analysis of engagement metrics and DISCERN scores for pictures								
	Number of likes - Total DISCERN score (pictures)	8	NA	NA	0.1791	NA	NA	-0.5275
	Number of comments - Total DISCERN score (pictures)	4	NA	NA	0.1670	NA	NA	-0.8330

Section 4A shows a significant association (p = 0.0041) between platform type and number of comments, underscoring YouTube's higher engagement levels compared to TikTok, Instagram, and Twitter. This variation suggests that user behavior is influenced by platform-specific features and content dynamics, making YouTube a preferred choice for active interaction.

Section 4B shows a significant variation in likes across platforms (p = 0.0047), highlighting YouTube's dominance in capturing user engagement. With its broader range of content and audience reach, YouTube provides opportunities for deeper interaction, reinforcing its position as a leading platform for content creators.

Section 4C implies the absence of a significant association between uploader type (individual vs. organization) and GQS or total DISCERN scores (p > 0.1) suggest that the credibility of the content is perceived similarly, irrespective of its source. This consistency indicates that users prioritize content quality over the uploader's identity when assessing informational value.

Section 4D Spearman's rank correlation analysis reveals weak and non-significant relationships between engagement metrics (likes, comments, views) and DISCERN scores for video content. These findings indicate that higher user interaction does not necessarily translate into higher informational quality, underscoring the need for creators to focus on content substance over engagement metrics.

In Section 4E, Pearson correlation analysis for picture-based content also shows no significant relationships between engagement metrics and DISCERN scores. Negative correlations for likes (-0.53) and comments (-0.83), though not significant, suggest that higher engagement might coincide with lower informational quality. This insight highlights the complexity of user interaction and its implications for content evaluation.

Table 4 shows that YouTube outperforms other platforms in terms of user engagement, with the highest median comments (33), likes (95), and view counts (12,496.5). These findings highlight YouTube's capacity to foster higher interaction levels, possibly due to its extensive user base and platform features that support detailed content. TikTok and Instagram, while showing engagement, trail behind YouTube, suggesting differing audience preferences and content-consumption habits.

**Table 4:** Comparison of comment counts, like counts and view counts on different platforms by median and interquartile range

Variables	Platform	Median	Lower quartile	Upper quartile
Comment counts	Instagram	3	1	40
	TikTok	5	1	10
	Twitter	2	1	2
	YouTube	33	11	61.5
Like counts	Instagram	19	16	39
	TikTok	49.5	23	178
	Twitter	9	7	66
	YouTube	95	49	230
View counts	Instagram	1160	140	2180
	TikTok	4160.5	1279	13600
	YouTube	12496.5	3823	24713

## Discussion

The current study underscores the critical role of social media platforms, particularly YouTube, in disseminating information about clubfoot. While YouTube exhibits broader reach and higher engagement compared to other platforms, the findings reveal that the quality and reliability of its content vary considerably. This study highlights that Arabic-language content related to clubfoot on four major platforms - YouTube, TikTok, X (formerly Twitter), and Instagram - generally falls within the poor to moderate range in terms of reliability and quality.

Both the DISCERN and Global Quality Score (GQS) scores used in this study indicate significant deficiencies in the quality of health-related posts across these platforms. This aligns with findings from previous research, such as Cassidy et al. (2016), who reported that online orthopedic information is typically of low quality and poor readability [12]. In the current study, the median DISCERN score of 2 suggests that most content provides limited useful information. This is consistent with a similar English-language study on YouTube videos about clubfoot, where a modified DISCERN score of 2.1±1.07 was reported, confirming comparable challenges in both Arabic and English-language content [13].

The GQS scores in this study, with a median score of 3, reflect a mix of fair to good quality content but also underline considerable room for improvement. These findings resonate with Hanna et al. (2021), who emphasized the importance of social media for information sharing and peer support among caregivers [10]. However, the current study uniquely addresses Arabic-language content, broadening the understanding of global disparities in online health information.

Notably, this study found no statistically significant correlations between engagement metrics (likes, comments, views) and content quality scores (GQS and DISCERN). This lack of correlation mirrors findings in previous studies, such as Oremule et al. (2019), which assessed rhinoplasty-related YouTube videos and concluded that engagement metrics do not reliably indicate information quality [14]. Similarly, Cassidy et al. (2016) pointed out that online orthopedic content often fails to meet high standards, reinforcing the necessity for clinicians to guide patients toward validated resources [12].

The uploader type in this study was not a determining factor in content quality, emphasizing the need for critical evaluation of all sources, regardless of their perceived credibility. This finding parallels prior conclusions that high engagement or professional affiliation alone is insufficient to ensure reliable health information [13,14].

The current study also complements Hanna et al. (2021), who highlighted the potential of social media for fostering communication between caregivers and healthcare providers [10]. By focusing on Arabic-language content, this research adds a regional perspective that was previously unexplored, emphasizing the universal challenge of ensuring reliable online health information.

The strengths of this study include its pioneering assessment of Arabic-language content on clubfoot across multiple platforms and the use of validated tools such as DISCERN and GQS. Additionally, it provides insights into the disconnect between engagement metrics and content reliability, a finding consistent with prior studies [13,14]. However, this study has several limitations that should be considered. First, the subjective nature of scoring tools like DISCERN and GQS may introduce variability despite efforts to standardize evaluations. Second, reliance on platform algorithms to identify content risks selection bias, as posts with low engagement or visibility were excluded, potentially limiting the comprehensiveness of the analysis and overrepresenting higher-quality or more popular posts. Additionally, the study’s focus on Arabic-language content, while valuable for understanding regional disparities, restricts the generalizability of findings to other linguistic or cultural contexts. The dynamic nature of social media further compounds this challenge, as content and engagement metrics evolve rapidly, potentially diminishing the relevance of these findings over time. Finally, platform-specific dynamics, such as TikTok’s short video format, may limit the depth of information conveyed, influencing content evaluation outcomes. Addressing these limitations in future research through longitudinal studies, inclusion of low-engagement posts, and the development of automated, objective scoring tools will be crucial for providing a more comprehensive understanding of online health information.

The authors recommend future studies to address these limitations by developing content creation guidelines and evaluating the effectiveness of educational interventions. These efforts are crucial to enhancing the quality of health information on social media, ensuring that caregivers and parents have access to accurate and reliable guidance. Aligning with the conclusions of prior studies, this research advocates for promoting high-quality content to meet the growing informational needs of caregivers in the digital era [12-14] they mention that this discussion is weak due to limitations is missing.

## Conclusions

In conclusion, this study underscores the critical need to improve the quality and reliability of Arabic-language content related to idiopathic clubfoot across social media platforms. While YouTube emerged as the platform with the highest levels of engagement among those analyzed, the overall quality of content, as evaluated using the DISCERN and GQS scores, was found to be generally poor to moderate. This finding highlights the significant gap in the availability of high-quality, reliable information about clubfoot for caregivers and patients seeking guidance. Importantly, the study revealed that there was no significant correlation between engagement metrics, such as likes, comments, and views, and the quality of the content. This suggests that high levels of user engagement do not necessarily equate to trustworthy or accurate information, which can be misleading for those relying on social media for medical advice.

These findings are particularly concerning, as they emphasize the need for greater scrutiny when evaluating online health information. The popularity of a post, as reflected in engagement metrics, should not be seen as an indicator of the quality or accuracy of the information presented. Instead, it is essential to focus on the educational value and factual accuracy of the content, especially when it pertains to the health and well-being of individuals. The lack of a direct correlation between engagement and content quality highlights the importance of health professionals and caregivers critically assessing online resources before using them as a basis for decision-making.

Overall, the study underscores the necessity for improving the quality of health-related content on social media platforms, particularly in the Arabic language. Moving forward, it is essential that content creators, healthcare professionals, and organizations work together to develop more reliable and informative resources for caregivers and patients. Promoting evidence-based, accurate, and accessible information can help ensure that individuals seeking guidance on complex health conditions like clubfoot can make informed decisions. Additionally, more rigorous quality evaluations and increased efforts to enhance content credibility are needed to ensure that online health information is both useful and trustworthy for the general public.
